# Setting Standards for Glaucoma Care

**Published:** 2012

**Authors:** Richard Wormald

**Affiliations:** Coordinating Editor: Cochrane Eyes and Vision Group (CEVG), International Centre for Eye Health, London School of Hygiene and Tropical Medicine, UK.

**Figure:**
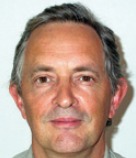
Richard Wormald

The UK National Institute for Health and Clinical Excellence (NICE) published guidelines on the diagnosis and management of open-angle glaucoma in 2009 **(www.nice.org.uk/CG85).** These are intended to set standards for practice in the UK's National Health Service (NHS)

As these guidelines are the only strictly evidence-based glaucoma guidelines available, however, they are a resource that can be accessed and used globally.

To produce evidence-based guidelines, questions are asked about which diagnostic tests to use, or which treatments to offer to particularl patients. High quality evidence is then systematically sought to answer those questions. When none is found, the consensus of the guideline development group is sought. When important gaps in the evidence base are found, recommendations for research are made.

## Why were the guidelines needed?

The process of developing guidelines is costly in terms of human resources, time, and money, so there has to be a good reason to produce them. To put it mildly, glaucoma care in the NHS was far from ideal; there were wide variations in practice and standards and, inevitably, in outcomes. Over-diagnosis and missed diagnoses were also widespread, as were over-treatment and under-treatment. Every year, about 1,500 people with glaucoma are registered as blind in the UK. This is despite the fact that medication is available to preserve existing vision and delay or prevent the progression to blindness for most patients.

## Limitations of the guidelines

It is important to remember that guidance is guidance, not a rigid protocol, and it is not comprehensive. It is ‘applicable to 80% of cases, 80% of the time’. In addition, as mentioned in other articles in this issue, eye drops are not always a feasible form of treatment in low- and middle-income countries, and therefore the guidelines may not be as widely applicable outside of the United Kingdom or Europe.

## The NICE quality standards for glaucoma

In addition to providing guidance to clinicians, it was also important to set standards for the delivery of glaucoma care **(http://guidance.nice.org.uk/QS7)**. This is applicable to the information systems, referral pathways, communication, staff management, etc. needed to provide patients with consistently high levels of care. These standards, although not easily achievable in poorly resourced settings, do give us some ideas about what needs to be in place in our eye care or health care systems if we want to reduce avoidable blindness from glaucoma. Primarily, what is needed is a reasoned and coordinated approach:

‘The quality standard for glaucoma requires that services should be commissioned from and coordinated across all relevant agencies encompassing the whole glaucoma care pathway, including primary, secondary and social care. An integrated approach to provision of services is fundamental to the delivery of high quality care to people with glaucoma. A local register of glaucoma-related conditions, organised according to diagnosis, could be used to facilitate such integration.’

Those most at risk of blindness from glaucoma are those in the most deprived circumstances, including material and educational poverty. For example, in the Caribbean, wealthy people with glaucoma can afford diagnosis and treatment while those on average incomes will have little chance of saving their sight.

**FROM THE FIELD: A response from Africa**

**Figure:**
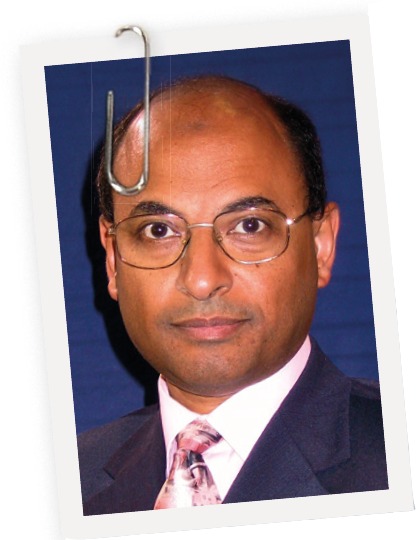
**Moustafa Yaqub** Professor of Ophthalmology, Assiut University, Egypt and Head of Ophthalmology Department, Royal Care International Hospital, Khartoum, Sudan.

There are two major problems in most African nations. The first is the severe lack of ophthalmologists trained in the diagnosis and treatment of glaucoma. The second is the absence of national public awareness programmes about glaucoma. Any standards formulated to improve the outcome of glaucoma management in Africa must address these two issues. The development of **awareness** among health care providers and the public is a must. The basic infrastructure needed for public awareness (TV, radio, newspapers), are available, but in the absence of governmental interest, they are under-utilised.

On the other hand, there is a real need for governmental involvement in the pricing of glaucoma medications. This is a global issue, but its impact is severely felt in under-developed countries. In some countries like Sudan, and for a variety of reasons, only a few drug companies have a market presence. As a result, patients do not have access to a good number of globally available glaucoma medications.

I think that, if the NICE guidelines are adapted by the WHO and used as the basis for a more comprehensive global guideline for glaucoma care, then more governments and health authorities can use them. More importantly, advocates for a better global care for glaucoma patients will then have a tool to use to convince policy makers to take action.

The huge challenge in poorer countries and emerging economies is to put in place the requirements for the ‘whole glaucoma pathway’. This is why preventing glaucoma blindness requires VISION 2020 programmes to achieve their highest potential by offering integrated and fully equipped services from primary to tertiary care. Thus the NICE standards are a remote ideal towards which all programmes should strive; the UK National Health Service (NHS) itself still has a long way to go.

For example, having local and/or national registers of glaucoma would help with the monitoring of standards, but this requires major infrastructural development. It would require a database that is secure in terms of information governance but also technically sustainable and fully, regularly, and automatically backed up. It must be able to maintain individual records over the 30-year natural history of the disease. However, basic beginnings can make an enormous difference. For example, on the island of Dominica, every person keeps an exercise book containing all their patient records; this is a useful solution when there are few resources.

